# News and Coming Events

**Published:** 1908-06-20

**Authors:** 


### News and Coming Events.

Dr. Gerald Leighton, M.D., F.R.S.E., has been appointed first Professor of Pathology and Bacteriology at the Royal Veterinary College, Edinburgh.

Mr. John Lentaigne has been elected President of the Royal College of Surgeons of Ireland for the ensuing year; Mr. Robert Woods has been elected Vice-President, and Sir Charles Cameron Secretary.

The annual dinner of the Association of British Postal Medical Officers will be held at the Whitehall Rooms, Hôtel Métropole, London, on Tuesday, July 14. The chair will be taken at 7.30 p.m. by Dr. G. A. Mason.

The King and Queen have been pleased to present signed photogravures of themselves to the King Edward VII. Sanatorium, Midhurst. The gifts have been hung in the entrance-hall of the institution.

The foundation-stone of the new Ramsgate General Hospital and Seamen's Infirmary was laid by Mrs. Charles Murray Smith, daughter of the former President, Mr. A. B. Warre, on June 11, Mr. L. W. Vaile presiding. The architects are Messrs. Woodd and Ainslie, and the contract price is just over £12,000.

The American Association of Medical Milk Commissions held its second annual meeting at Chicago on June 1, under the Presidency of Dr. Henry L. Coit. This Association is composed of physicians from various parts of the United States, who are engaged in milk commission work, and professional sanitarians and hygienists, who are connected with the pure-milk movement.

Dr. Edwin Rickards, J.P., M.B., F.R.C.P., consulting physician to the General Hospital, Birmingham, died on June 11 at Edgbaston. Dr. Rickards was sixty-six years old at the time of his death. He was educated at Oxford University and University College, London, and qualified in 1870, having taken his M.A. degree five years previously. In 1870 he also obtained the fellowship of the Royal College of Surgeons of England, while in 1886 he was elected a Fellow of the Royal College of Physicians of London. Dr. Rickards, before his appointment as physician to the General Hospital, Birmingham, had held a number of junior medical posts at that institution, and also at University College Hospital. He held, in addition, the appointment of honorary physician to the General Institution for the Blind. He was an occasional contributor to current medical literature.

The annual election to the Council of the Royal College of Surgeons of England will take place on July 2. The retiring members of the Council are Mr. A. Pearce Gould, of Middlesex Hospital, Mr. J. Ward Cousins, of Portsmouth, and Mr. W. Harrison Cripps, of St. Bartholomew's Hospital. Prof. Howard Marsh, of Cambridge, has also resigned his seat on the Council. There are thus four vacancies to be filled. Mr. J. Ward Cousins does not intend to offer himself for re-election, and the following is the full list of candidates: Mr. Pearce Gould and Mr. Harrison Cripps, who offer themselves for re-election; Mr. George Eastes, who will stand as "a representative of general practitioners"; Mr. W. Arbuthnot Lane, of Guy's Hospital; Mr. Bilton Pollard, of University College Hospital; Mr. C. B. Lockwood, of St. Bartholomew's Hospital; Mr. G. F. Haslam, of Birmingham; and Mr. J. Lynn Thomas, C.B., of Cardiff.

Dr. Francis R. Hill, M.B., C.M., Glasgow, has been appointed surgeon-in-charge of the new eye department of the Cumberland Infirmary, Carlisle.

The Earl of Crewe will unveil the statue of the Queen at the London Hospital on Friday, July 10, at 4 p.m. The prize distribution to the students and probationers of the Medical College will take place at 3.30 on the same afternoon.

Dr. Fulgence Raymond, Professor of Neurology at the Salpêtrière Hospital, Paris, has consented to hold a clinique at 11 a.m. on Friday, June 26, in the Physiology Theatre of Guy's Hospital. He will demonstrate two or three nervous cases, in order to illustrate the methods he employs at his famous Tuesday lectures at the Salpêtrière.

The Court of the University of Edinburgh has decided not to accept the scheme presented by the Scottish Association for the medical education of women. The Court considers that it does not possess, and sees no definite prospect of acquiring, resources for providing separate accommodation for the medical education of women; and, further, it does not see its way to approve of mixed classes in the Faculty of Medicine.

Congress has granted a pension of $1,500 a year to the widow of Dr. Jesse W. Lazear, who died of yellow fever experimentally induced, and the same amount to the widow of Major James Carroll, who died of heart disease, resulting from an attack of yellow fever also experimentally induced. Both were members of the Army Commission, under the late Major Reed, which studied yellow fever in Cuba and confirmed Finlay's theory that the disease is spread through the agency of mosquitoes.

A Parliamentary Paper was issued on June 16 showing the number of deaths in India for a series of years from cholera, small-pox, and plague. In 1906 the deaths were: From cholera, 690,519; from small-pox, 109,583; and from plague, 300,355; or 3,053.7, 484.6, and 1,328.3 respectively per million of the population. In 1896 the figures were: Cholera, 469,679; small-pox, 136,605; and plague, 2,219; the respective ratios per million of population being 2,208.5, 642.3, and 10.4. In 1877 the deaths from cholera numbered 624,675, the ratio per million of population being 3,433.6; while the deaths in that year from small-pox totalled 183,734, the ratio being 1,009.9.

On June 16 in the House of Commons Mr. Lee asked the Prime Minister whether the official announcement that the Imperial Cancer Fund was supported by the Government implied that this fund was supported and financed, mainly or partially, out of public funds; whether he was aware that urgent appeals for further financial assistance to this Fund were being publicly made by its treasurer; and whether the Government could see its way to respond to this appeal by an adequate grant from the Exchequer? Mr. Asquith replied that when he said that the Imperial Cancer Research Fund was supported by the Government, what he meant was that the Government accorded it practical assistance in the collection and transmission of information and specimens and in defraying the incidental expenses connected with this work. The Fund was not directly financed out of public funds, and he doubted, though the question was rather one for the Chancellor of the Exchequer, whether it would be possible to give it the exceptional favour of a grant from the Exchequer.

The Lord Chief Justice, who presided over the thirty-fifth annual meeting of the National Health Society, said that an extraordinary change had come over public opinion since the Society's formation. In the past year thirty of the Society's students had gained appointments as sanitary inspectors, health visitors, relieving officers, etc. This showed what a demand there was for trained and qualified women. The Society pointed out a useful way in which ladies who needed employment could obtain it. Medical men had dwelt on the almost hopeless ignorance of many poor mothers as to the feeding and bringing up of young children, and he had had extraordinary evidence of this in cases brought before him as a Judge. The work of health visitors was, therefore, greatly needed. The Archdeacon of London commended the Society for constantly revising its publications in the light of fresh medical knowledge. Professor Sims Woodhead said that the work of teaching people how to avoid disease was still left too much to the few who had come into contact with the evils caused by ignorance. There had been a great fall in the general death-rate, but not in that of infants, largely owing to the crass ignorance among those who ought to be best informed—the mothers, If as much time and money were devoted to prevention as to cure, many of our hospitals might be shut, and there would be plenty of funds available for those that remained.

A Bill designed to eradicate bovine tuberculosis from New York State and to restrict the use of the tuberculin test has been passed by the Senate. According to the *Times*, it is urged by the supporters of the Bill that great losses have been suffered by dairymen through the indiscriminate destruction of healthy animals condemned as unfit after the application of tuberculin. While the value of tuberculin is acknowledged when properly administered, the test has been condemned because it has been misused, and its effect has been misjudged, and there are charges that fraudulent substances have been substituted for the genuine fluid, with consequent wrong diagnosis. The Bill provides for physical examination; for the adoption of the Danish system of sanitation and segregation; and for the pasteurisation of the milk of animals suffering from localised tuberculosis.

At a meeting of the Royal Sanitary Institute on June 13, a paper on "The Pasteurisation of Milk" was read by Professor H. R. Kenwood. Although he admits that the use of sterilised and boiled milk occasionally harms a child, Professor Kenwood maintains that it could not be in the public interest to discourage the use of such milk in face of the overwhelmingly greater dangers of raw milk. While everything that is possible should be done to bring about improved conditions at the farms, he strongly advocates the pasteurisation of the public milk supply, which affords, in his opinion, a readily available means of bringing about a reduction of infantile sickness and mortality. In the afternoon Mr. and Mrs. Titus Barham gave a garden party at Sudbury Park, Wembley, to meet the Chairman and members of the Institute. The guests were conducted over the model dairies and farm, and the Walker-Gordon laboratories. The laboratories are intended to solve as far as possible the problem of pure milk supply and the scientific feeding of infants. The milk, within four minutes of being obtained from the cow, is reduced from about 90° to 37° F., and is then packed in hermetically sealed bottles and placed in cold storage to await delivery. Milk suitable to infants of varying age and constitution is also made up in the laboratories at the dairy according to medical prescriptions, and the resulting fluid is placed in sterilised bottles so constructed that they may be used as feeding bottles direct.

The Royal London Ophthalmic Hospital, City Road, has been compelled to issue a notice that unless £5,000 is immediately forthcoming the committee will be forced to close some thirty beds. This hospital relieves daily on an average four hundred out-patients and about one hundred in-patients. It has a warm recommendation as to efficiency, economy, and management from the Council of the King's Fund.

By the death of Dr. Thomas Lambert Hinton, which took place at St. Leonards-on-Sea on June 14, the Royal College of Surgeons loses its oldest member. Dr. Hinton celebrated his hundredth birthday on May 1 last. In 1829 he joined the 1st Bengal Fusiliers, and served with that regiment as an assistant surgeon until the year 1846. Meanwhile, in 1833, he became a member of the Royal College of Surgeons and a licentiate of the Society of Apothecaries. On his way home from India he was wrecked off Cape Finisterre, and whilst in the water he swam with a child that had been flung to him for some time before being rescued. Dr. Hinton was educated at Oxford, Paris, and London, and at one time held the post of surgeon to the Reading Dispensary.

